# Crinone Gel for Luteal Phase Support in Frozen-Thawed Embryo Transfer Cycles: A Prospective Randomized Clinical Trial in the Chinese Population

**DOI:** 10.1371/journal.pone.0133027

**Published:** 2015-07-29

**Authors:** Yang Wang, Yaqiong He, Xiaoming Zhao, Xiaowei Ji, Yan Hong, Yuan Wang, Qinling Zhu, Bin Xu, Yun Sun

**Affiliations:** 1 Center for Reproductive Medicine, Ren Ji Hospital, School of Medicine, Shanghai Jiao Tong University, Shanghai, China; 2 Shanghai Key Laboratory for Assisted Reproduction and Reproductive Genetics, Shanghai, China; Center for Human Reproduction, UNITED STATES

## Abstract

To compare Crinone vaginal progesterone gel with intramuscularly injected progesterone for luteal phase support in progesterone-supplemented frozen-thawed embryo transfer (FET) cycles, a randomized prospective study of patients qualified for FET was conducted between September 2010 and January 2013 at a hospital in Shanghai, China. From the day of transformation into secretory phase endometrium (day 0), Crinone vaginal gel (90 mg/d) was administered to patients in the Gel Group, while progesterone (40 mg/d) was injected intramuscularly in patients in the Inj Group (n = 750 per group). All patients received oral dydrogesterone (20 mg/d) and estradiol valerate (4–8 mg/d). Day 3 embryos with the highest pre-frozen scores were transferred to patients in the two groups and the clinical outcomes compared. This study comprised 1,500 cycles (750 in each group). Twenty-nine cycles in the Gel Group and 24 in the Inj Group were withdrawn. There were no significant differences between groups in age, endometrial thickness, endometrial preparation time or number of embryos transferred. No significant differences were observed between the Gel Group and Inj Group in the rates of live birth (32.6% vs. 31.7%, *P* = 0.71), clinical pregnancy (40.1% vs. 40.6%, *P* = 0.831), implantation (25.8% vs. 25.3%, *P* = 0.772), abortion (16.3% vs. 18.3%, *P* = 0.514) or ectopic pregnancy (2.8% vs. 4.4%, *P* = 0.288). Multivariate logistic regression analysis revealed that the odds ratios (95% confidence intervals) for the rates of live birth, clinical pregnancy, abortion and ectopic pregnancy (Gel Group relative to Inj Group) were 1.036 (0.829–1.295), 0.971 (0.785–1.200), 0.919 (0.595–1.420) and 0.649 (0.261–1.614), respectively. Our study revealed that using Crinone vaginal gel in FET cycles achieved similar pregnancy outcomes to intramuscular progesterone, indicating that vaginal gel is a viable alternative to intramuscular injection.

**Trial Registration:**

Chinese Clinical Trial Registry ChiCTR-TRC-14004565

## Introduction

The use of frozen-thawed embryo transfer (FET) to achieve a successful pregnancy was first reported in 1983, and since then, FET has been applied widely in assisted reproduction [1], achieving an increased cumulative pregnancy rate while reducing patient costs. Improvements in vitrification technology have allowed more mitotic embryos and blastocysts to be obtained [2] and higher clinical pregnancy and live birth rates to be achieved [3], compared with the results of conventional cryopreservation by slow freezing. Endometrial preparation can be achieved by hormone replacement during FET cycles, and is favored by an increasing number of clinicians as it is simple to apply and reduces both the monitoring frequency and cycle cancellation rate, thereby providing more convenient working arrangements.

Progesterone plays an important role in endometrial-embryo synchrony and the maintenance of early pregnancy [4]. FET is used mainly in women who have failed to become pregnant following fresh embryo transfer cycles, whose endometrium is not suitable for fresh embryo transfers, or who are at high risk of ovarian hyperstimulation syndrome. The corpus luteum does not form endogenously in FET cycles due to the absence of ovulation; therefore, secretory transformation of the endometrium prior to embryo transfer and post-transfer maintenance of normal embryonic development are totally dependent on exogenous progesterone supplementation. There are currently three possible routes of progesterone administration: oral, intramuscular and vaginal. Although oral medication is convenient, bioavailability is low due to the hepatic first-pass effect, and secretory transformation of the endometrium is not achieved [5]. Intramuscular progesterone administration is frequently used in clinical practice as it is inexpensive and achieves both a high serum level and a stable clinical pregnancy rate. However, intramuscular progesterone administration is painful, requires daily injections, and may be inconvenient due to the commute required between the home and hospital. Furthermore, administration via the intramuscular route may lead to marked inflammation at the injection site, resulting in symptoms such as allergic reactions or local panniculitis, which may progress to abscesses [6]. The vaginal route of progesterone administration is convenient and achieves a stable endometrial concentration with low serum levels, reducing the risks of systemic adverse effects [7]. Nevertheless, debate remains concerning the clinical outcomes of FET cycles that use vaginally administered progesterone.

No consensus has yet been reached as to whether the route of administration of progesterone support affects the clinical outcome of FET cycles. Moreover, no randomized controlled trial with a large sample size has been conducted to compare the clinical outcomes of FET cycles between vaginal and intramuscular progesterone administration. Therefore, to provide a scientific basis for the clinical use of vaginal gel, this prospective randomized study was carried out to compare the live birth rates yielded from FET cycles between patients administered progesterone as a vaginal gel and those administered progesterone by intramuscular injection.

## Materials and Methods

### Study design

This was a single-center, prospective, randomized, two-arm trial. The protocol was approved by the Ethics Committee of Renji Hospital, Shanghai Jiaotong University School of Medicine (Ethical Review No.067, 2010). This study was registered at the Chinese Clinical Trial Registry (Registration No.: ChiCTR-TRC-14004565).

The study comprised 1,500 progesterone-supplemented FET cycles conducted between September 2010 and January 2013 at the Reproductive Center of Shanghai Renji Hospital. Patients were randomized into two groups, to receive progesterone either as Crinone vaginal gel (Gel Group) or by intramuscular injection (Inj Group). All patients provided written informed consent voluntarily.

### Sample size calculation

We hypothesized that the live birth rate of patients in the Gel Group was 10% higher than that of patients in the Inj Group (40% vs. 30%). A 90% statistical power (α = 0.01, ratio = 1:1) was achievable by the inclusion of 675 cycles in each group. A total of 1,500 cycles (750 cycles in each group) were included in the final enrollment to allow for a withdrawal rate of approximately 10%.

### Subjects

Subjects were enrolled between September 2010 and January 2013 at the Reproductive Center of Shanghai Renji Hospital.

The inclusion criteria were: patients aged between 20 and 40; day 3 frozen embryos; and an endometrial thickness ≥7 mm on the secretory transformation day.

The exclusion criteria were as follows: patients with uterine disorders (including uterine malformations such as unicornuate uterus, bicornuate uterus, septate uterus and uterus duplex), adenomyosis, submucous myoma or intrauterine adhesions; patients with a history of natural abortions (including biochemical pregnancies) or embryo transplant failures (including biochemical pregnancies) on more than three occasions; patients taking drugs or therapies that may affect reproductive or metabolic functions, such as anti-diabetic drugs, anti-hypertensive drugs (including diazoxide, ACEI inhibitors and calcium channel blockers), Chinese herbal medicines and acupuncture; patients with endometrial thickness <7 mm on the secretory transformation day; and patients who were unable to comply with the study protocol (S1 Text).

### Randomization

A computer-based random allocation table was generated by researchers who were blind to this study. The table randomized the 1,500 cycles into the two groups using 1,500 numbers with a ratio of 1:1 (e.g.: random number 1 was allocated into the Gel Group). The researchers and patients were informed of the grouping results on day 0. Masking or blinding was not possible in this study.

### Treatment

All the enrolled patients had taken estradiol valerate (Progynova, Bayer, Germany) 4 mg/d orally for 10 days since the third day of menstruation. The dose of estradiol valerate was subsequently increased to 6–8 mg/d in cases where the B ultrasound images showed the endometrial thickness to be <7 mm, and the treatment was continued for 7–14 days until the endometrial thickness was ≥7 mm. Those patients whose endometrial thickness remained <7 mm after this dose titration were excluded. Progesterone was used to assist secretory transformation of the endometrium. On day 0 of luteal support, Crinone vaginal gel (Merck Serono, Switzerland) was administered at a dose of 90 mg/d to patients in the Gel Group, while progesterone (Shanghai General Pharmaceutical Co., Ltd., China) was injected intramuscularly at a dose of 40 mg/d into patients in the Inj Group. Dydrogesterone (20 mg/d; Duphaston, Abbott Healthcare, USA) and estradiol valerate (4–8 mg/d) were given orally to all patients from day 0 of luteal support. Patient withdrawal on the day of embryo transfer (day 3) accounted for 29 cycles in the Gel Group and 24 cycles in the Inj Group. Day 3 embryos with the highest pre-frozen scores were transferred, and the same medication regimens were continued for 14 days after embryo transfer. The number of embryos transferred was based on the criteria of the Ministry of Health, and was a maximum of 2 for women aged under 35 years undergoing embryo transfer for the first time, and 2–3 in women aged 35 years or older undergoing retransplantation.

### Follow-up of the pregnancy outcome

The presence of biochemical pregnancy was confirmed by measurement of serum β-hCG levels 14 days after the transfer. The treatment regimen (i.e. dydrogesterone, estradiol valerate and either Crinone vaginal gel or intramuscular progesterone injection) was continued without change in women testing positive for β-hCG (5 IU/L). Clinical pregnancy was determined by the presence of gestational sacs in B ultrasound images 5 weeks post-transfer. Estradiol valerate was stopped 6 weeks post-transfer, Crinone vaginal gel/intramuscular progesterone was stopped 8 weeks post-transfer, and dydrogesterone was stopped 10 weeks post-transfer. Details of the delivery and infant health status were obtained during follow-up through letters or telephone calls.

### Adverse events

The occurrences of any adverse events such as bleeding, itching, or inflammation at the injection sites were recorded. Patients were able to withdraw from the study if severe adverse events occurred.

### Statistical analysis

Statistical Product and Service Solutions (SPSS) 18.0 software was used for analysis. Data are expressed as mean ± standard deviation (SD) or percentage (%). Enumeration data were analyzed by the *χ* ^*2*^ test. Measurement data were tested for normality, and analyzed by either the Student’s *t*-test (normally distributed data) or Mann-Whitney U test (non-normally distributed data). Univariate and multivariate logistic regression analysis were performed, with adjustment for potential covariates (age, endometrial thickness, endometrial preparation time, number of embryos transferred, number of high-quality embryos transferred and embryo recovery rate), in order to calculate odds ratios (ORs) and corresponding 95% confidence intervals (95%CIs) for clinical pregnancy rate, abortion rate, ectopic pregnancy rate and live birth rate for the Gel Group relative to the Inj Group. A P-value less than 0.05 was considered statistically significant.

## Results

### Baseline characteristics of the patients

**Fig 1:**
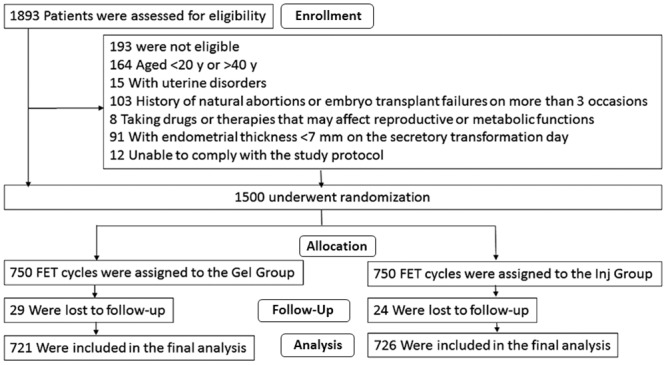
Study protocol.

A diagram showing participant flow through the study, including details of patient eligibility, reasons for exclusion, treatment group allocation, loss to follow-up and number included in the final analysis, is shown in Fig 1.

There were no statistically significant differences between the Gel Group and Inj Group in patient age, endometrial thickness, endometrial preparation time, number of embryos transferred, number of high-quality embryos transferred and embryo recovery rate (Table 1).

**Table 1:** Comparison of the baseline characteristics of the patients included in the analysis. Data are presented as the mean ± standard deviation.

	Gel Group *N* = 721	Inj Group *N* = 726	*P* value
**Age (years)**	30.6 ± 4.0	30.8 ± 4.3	0.331
**Endometrial thickness (mm)**	8.8 ± 1.0	8.8 ± 1.2	0.956
**Endometrial prep aration time (days)**	18.3 ± 4.0	18.3 ± 4.0	0.986
**Number of embryos transferred (*n*)**	2.07 ± 0.44	2.1 ± 0.5	0.144
**Number of high-quality embryos transferred (*n*)**	1.23 ± 0.8	1.23 ± 0.84	0.821
**Embryo recovery rate (%)**	92.7 ± 16.9	93.8 ± 15.6	0.190

### Clinical outcomes

After the exclusion of cycles withdrawn from the study, the primary analysis included 721 cycles in the Gel Group and 726 cycles in the Inj Group. As shown in Table 2, there were no statistically significant differences between the Gel Group and Inj Group in the rates of live births (32.6% vs. 31.7%, *P* = 0.710), clinical pregnancy (40.1% vs. 40.6%, *P* = 0.831), implantation (25.8% vs. 25.3%, *P* = 0.772), abortion (16.3% vs. 18.3%, *P* = 0.514) and ectopic pregnancy (2.8% vs. 4.4%, *P* = 0.288). Both univariate and multivariate logistic regression analysis revealed that the odds ratios (Gel Group vs. Inj Group) for live birth rate, clinical pregnancy rate, implantation rate (didn’t conform to the binomial distribution and thus was not included in multivariate analysis), abortion rate and ectopic pregnancy rate were not significantly different from unity (Table 2).

**Table 2:** Clinical outcomes in the two groups and odds ratios (Gel Group vs. Inj Group) for clinical outcomes. Data are presented as % (*n*/*N*) or as odds ratio (OR) with 95% confidence interval (CI).

	Gel Group *N* = 721	Inj Group *N* = 726	*P* value	Univariate OR (95%CI)	Multivariate OR (95%CI)
**Clinical pregnancy rate**	40.1% (289/721)	40.6% (295/726)	0.831	0.977 (0.792–1.206)	0.971 (0.785–1.200)
**Implantation rate**	25.8% (384/1490)	25.3% (386/1525)	0.772	1.025 (0.870–1.207)	—*
**Abortion rate**	16.3% (47/289)	18.3% (54/295)	0.514	0.867 (0.564–1.332)	0.919 (0.595–1.420)
**Ectopic pregnancy rate**	2.8% (8/289)	4.4% (13/295)	0.288	0.618 (0.252–1.513)	0.649 (0.261–1.614)
**Live birth rate**	32.6% (235/721)	31.7% (230/726)	0.710	1.043 (0.836–1.300)	1.036 (0.829–1.295)

*, implantation rate didn’t conform to the binomial distribution and thus was not included into multivariate analysis.

#### Adverse effects

Twenty-nine cycles were withdrawn from the Gel Group, 24 due to embryo recovery failure and 5 due to vaginal bleeding or itching that necessitated a change to intramuscular progesterone injection. Twenty-four cycles were withdrawn from the Inj Group, 16 due to embryo recovery failure and eight due to inflammation at the injection sites that necessitated a change to a vaginal gel (these symptoms were alleviated after therapy with hot compresses). No other adverse events occurred in the women involved in this study.

## Discussion

This prospective randomized study was designed to compare the clinical effects of using Crinone vaginal progesterone gel in FET cycles with those of progesterone supplementation by intramuscular injection. Analysis of the primary outcomes indicated that there were no statistically significant differences in the rates of live births, clinical pregnancy, implantation, abortion and ectopic pregnancy between the Gel Group and the Inj Group.

There has been debate as to whether the live birth rate achieved following use of Crinone vaginal gel during FET cycles is comparable to that obtained following intramuscular administration of progesterone. Several studies of *in vitro* FET cycles [8–11] have reported that similar results are achieved when progesterone is administered via vaginal gel and intramuscular injection. In contrast, another report [12] showed that luteal phase support with a vaginal gel produced significantly higher live birth rates than intramuscular progesterone administration. A prospective study by Gibbons et al. [13] demonstrated that intramuscular progesterone replacement (100 mg/d, *n* = 18) was as effective as vaginal progesterone replacement using a polycarbophil gel preparation (90 mg twice daily, *n* = 54) at producing clinical and ongoing pregnancies within their donor egg program. Jobanputra et al. [14] reached a similar conclusion based on their prospective study showing that 8% Crinone (100 mg/day, *n* = 42) produced the same clinical and ongoing pregnancy rates as intramuscular progesterone (90 mg/day, *n* = 44) in women who required complete progesterone replacement. However, the statistical powers of these two early prospective studies, as well as that conducted by Toner [15], were poor due to the limited sample sizes. A retrospective study by Berger et al., conducted as part of an oocyte donation program [16], reported no statistically significant difference in live birth rate between the two administration routes, whereas a recent retrospective study by Kaser et al. found thatthe odds of clinical pregnancy and live birth were lower for day 3 cryopreserved embryo transfer cycles with 8% Crinone luteal support than for those with intramuscular progesterone support [17]. Kaser et al. hypothesized that the differences in pregnancy outcome between these two administration routes may be due to the local high concentration of vaginal gel inside the endometrium; thus, a delay in the administration of the Crinone vaginal gel may avoid early closure of the implantation “window” during FET cycles. However, a recent study by Shapiro et al. reported no difference in clinical outcome between the two routes of progesterone administration, despite the absence of a delay in the vaginal gel treatment [18]. These clinical trials are all evidence-based retrospective studies with many confounding factors between the two groups, emphasizing the need for higher-quality prospective studies in order to reach a more reliable conclusion. In the present study, no significant differences were observed between groups in pregnancy outcomes, including the rates of clinical pregnancy and live birth. It is notable that in our study, Crinone gel and intramuscular progesterone were both initiated on day 0, and all patients had equivalent exposure to dydrogesterone and estradiol valerate before the transfer was carried out. Thus, our findings would not appear to be consistent with the proposal of Kaser et al. [17].

It has been reported that multiple factors affect the synchronization between the embryonic stage and endometrial receptivity, including the application of estrogen, endometrial thickness, luteal support before embryo transfer, embryo quality, embryo number and FET technology [19]. In the randomized prospective trial reported here, no differences were observed between women in the Gel Group and those in the Inj Group with regard to age, endometrial thickness, endometrial preparation, and the number and quality of embryos transferred. Thus, confounding factors with the potential to influence the clinical outcome of FET did not differ between the participants in the two groups. Furthermore, the lack of significant difference between groups in clinical outcomes was supported by multivariate logistic regression analysis in which adjustments were made for these covariates. Additionally, since dydrogesterone is highly selective for progesterone receptors and has an immunomodulatory effect that may induce protection of the pregnancy, all patients enrolled in this study were medicated with an equal dose of this compound [20]. Thus, our results show no significant differences between the two groups in the live birth rate and other outcome measures (clinical pregnancy rate, implantation rate, abortion rate and ectopic pregnancy rate). Furthermore, our study had a relatively large sample size (721 cycles in the Gel Group and 726 cycles in the Inj Group), and its results are validated by a statistical power of 90% (α = 0.01).

Exogenous progesterone supplementation in FET cycles is often required until 12 weeks of gestation, a longer period than that required for fresh embryo transfer cycles. Although the use of intramuscular progesterone lowers patient costs, it requires daily injections and is associated with a number of drawbacks; for example, hospital visits may be required for treatment, and inflammatory responses may occur at the injection site [6]. In this study, eight women in the Inj Group were switched to vaginal gel administration due to inflammation at the injection sites. Importantly, our study shows that Crinone vaginal gel and intramuscular progesterone are equivalent in terms of pregnancy outcomes, with the vaginal gel route offering the additional advantages of improved administration convenience, comfort and compliance [8,21,22].

The present study utilized once-daily administration of Crinone gel (90 mg/d), whereas several previous studies have used a twice-daily dosing regimen [13,16–18]. Interestingly, Alsbjerg et al. [23] reported that increasing the dose of Crinone gel during the FET cycle from 90 mg/d (single administration) to 180 mg/d (twice daily administration) yielded a significantly lower early abortion rate and significantly higher rates of clinical pregnancy and live births. Thus, it is possible that a higher rate of live births would have been observed in the present study if a twice-daily dosing regimen had been employed in the Gel Group. However, it should also be noted that the live birth rate of the Gel Group in the present study (40%) was, in general, not inferior to rates of 24% [17], 39% [24] and 34% [25] reported previously in studies investigating twice-daily administration of Crinone gel. Therefore, additional prospective, randomized, controlled, blinded trials are merited to determine the optimal dosing regimen for Crinone vaginal gel.

The present study has several limitations. First, the study was conducted in a single center; thus, further large-scale prospective cohort studies are required to validate our findings and increase the generalizability of our conclusions. Second, it was not possible to conduct a blinded study; however, confounding factors were minimized where possible. Third, the study did not contain a control comparator group consisting of patients administered oral dydrogesterone and estradiol valerate only, precluding assessment of whether additional progesterone supplementation with either Crinone gel or intramuscular injections resulted in improved pregnancy outcomes in patients receiving dydrogesterone. Fourth, the incidence of vaginal bleeding or spotting in each group before and after the pregnancy test was not assessed as an outcome measure. Fifth, the doses of Crinone vaginal gel and intramuscular progesterone used in the present study were lower than those used in previous studies conducted in the USA, limiting the comparison of our protocols to those commonly used in the USA.

Our results indicate that the live birth rates were similar when progesterone supplementation of FET cycles was achieved using vaginal gel and intramuscular progesterone, and that Crinone vaginal gel does not affect other pregnancy outcomes. Since the use of intramuscular progesterone injections for endometrial preparation in progesterone-supplemented FET cycles has disadvantages in terms of convenience and patient tolerance, the results of this study suggest that Crinone vaginal gel is an effective and tolerable alternative during the induction of secretory transformation of the endometrium.

## Supporting Information

S1 Text Study protocol. (DOCX)

S2 Text Checklist. (DOC)
