# Supplementary material for: Crinone Gel for Luteal Phase Support in Frozen-Thawed Embryo Transfer Cycles: A Prospective Randomized Clinical Trial in the Chinese Population
Source: PLoS One. 2015 Jul 29;10(7):e0133027. doi: 10.1371/journal.pone.0133027 (PMC4519178; doi:10.1371/journal.pone.0133027)
Supplement: S1 Text — (DOCX) [file pone.0133027.s001.docx]

**A comparison of live birth rates between** **sustained release vaginal progesterone gel and intramuscular injection of progesterone in hormone replacement frozen-thawed embryo transfer cycles: a randomized prospective study in Chinese population**

**Table of Contents**

[1 Composition of committee 2](#_Toc406238429)

[1.1 Protocol committee 2](#_Toc406238430)

[1.2 Monitoring committee 2](#_Toc406238431)

[2 Background 2](#_Toc406238432)

[3 Objective 4](#_Toc406238433)

[4 Flow chart and visit 4](#_Toc406238434)

[4.1 Flow chart 4](#_Toc406238435)

[4.2 Screening visit 5](#_Toc406238436)

[4.3 Visit on the day of endometrial secretory transformation 6](#_Toc406238437)

[4.4 Visit on the day of embryo transfer 6](#_Toc406238438)

[4.5 Visit on the day of determination of biochemical pregnancy 6](#_Toc406238439)

[4.6 Visit on the day of evaluation of clinical pregnancy 7](#_Toc406238440)

[4.7 Visit of delivery outcomes 7](#_Toc406238441)

[5 Design 7](#_Toc406238442)

[5.1 Overall design 7](#_Toc406238443)

[5.2 Randomization 7](#_Toc406238444)

[5.3 Protocol of frozen embryo hormone replacement 8](#_Toc406238445)

[5.4 Endometrial secretory transformation and luteal phase support 8](#_Toc406238446)

[5.5 Pregnancy outcome follow-up 9](#_Toc406238447)

[5.6 Endpoint 9](#_Toc406238448)

[6 Subjects selection 9](#_Toc406238449)

[7 Physical examination 10](#_Toc406238450)

[8 Transvaginal ultrasound examination 10](#_Toc406238451)

[9 Outcome measures 10](#_Toc406238452)

[10 Duration 10](#_Toc406238453)

[11Data statistics 11](#_Toc406238454)

[11.1 Estimation of sample size 11](#_Toc406238455)

[11.2 Statistical methods 12](#_Toc406238456)

[12 Adverse event reports 12](#_Toc406238457)

[12.1 Risks and discomfort 12](#_Toc406238458)

[12.2 Definition of adverse event 13](#_Toc406238459)

[12.3 Adverse event log 13](#_Toc406238460)

[12.4 Causality and severity assessment 14](#_Toc406238461)

[12.5 Immediate action following a serious adverse events 14](#_Toc406238462)

[13Ethics 15](#_Toc406238463)

[14References 15](#_Toc406238464)

[Annex 1: Informed consent form 16](#_Toc406238465)

[Annex 2: Case report form (CRF) 16](#_Toc406238466)

# 1 Composition of committee

## 1.1 Protocol committee

| Name | Title | Units |
| --- | --- | --- |
| Sun Yun | Principal investigator | Reproductive Medicine Center of Renji Hospital Shanghai Jiaotong University School of Medicine |
| Zhao Xiaoming | Investigator | Reproductive Medicine Center of Renji Hospital Shanghai Jiaotong University School of Medicine |
| Hong Yan | Investigator | Reproductive Medicine Center of Renji Hospital Shanghai Jiaotong University School of Medicine |
| Wang Yao | Investigator | Reproductive Medicine Center of Renji Hospital Shanghai Jiaotong University School of Medicine |
| Xu Bing | Investigator | Reproductive Medicine Center of Renji Hospital Shanghai Jiaotong University School of Medicine |
| Ji Xiaowei | Investigator | Reproductive Medicine Center of Renji Hospital Shanghai Jiaotong University School of Medicine |

## 1.2 Monitoring committee

| Name | Duty | Units |
| --- | --- | --- |
| Zhu Wenting | To monitor protocol execution and clinical data | Shandong University School of Medicine |
| Lu Qi | To monitor safety and ethic | Ethics committee of Renji Hospital Shanghai Jiaotong University School of Medicine |

# 2 Background

With development and improvement of assisted reproductive technology, good quality embryos in most of patients can be frozen in vitro fertilization cycle. Frozen-thawed embryo transfer (FET) is an important technology derived from assisted reproductive field and has many advantages such as retrieving oocyte once with multiple transferring, increasing cumulative pregnancy rates, reducing multiple pregnancy rates and alleviating the pain due to ovarian hyperstimulation and oocyte retrieval and economic burdens. Currently, FET is mainly used in fresh cycle to prevent ovarian hyperstimulation syndrome, endometrium not suitable for transfer or fresh embryo transferred on the cycle of oocyte retrieval without pregnancy and so on. Hormone replacement FET cycle is widely applied due to monitoring simple, flexible control, low cycle cancellation rate and other advantages. Exogenous progesterone, however, is required to induce endometrial secretory transformation to receive embryo implantation and develop as hormone replacement cycle no follicular development and ovulation. Progesterone injections in inducing endometrial secretory transformation as luteal phase support is commonly used in China on account of cheap and stable pregnancy rate, but it has some disadvantages such as injection pain, abscess and panniculitis on injection site, allergic reaction, visiting hospital frequently and others ^[1]^. Sustained release vaginal progesterone gel is a micronized natural progesterone vaginal preparation, reducing local adverse reactions and having a better compliance when compared with intramuscular injection. After the first pass effect of the uterus, it spreads to cervix and corpus uteri and accumulates to a high concentration in uterus locally. Due to biological sustained release administration, progesterone concentration in the endometrium is also very stable with a lower proportion absorbed into the bloods, which effectively reduce the risk of systemic side effects ^[2]^.

It has been reported in recent years that there were no significant differences in live birth rate and clinical pregnancy rate between sustained release vaginal progesterone gel and intramuscular progesterone as luteal phase support in fresh embryo transfer cycles ^[3-6]^. And Jobanputra K, et al. performed endometrial biopsy and histologically confirmed that sustained release vaginal progesterone gel produced the same endometrial development as intramuscular progesterone in the artificial cycle ^[7]^. But, currently, there is no relevant randomized controlled trial (RCT) to compare live birth rates in hormone replacement FET with different routes of progesterone supplementation.

# 3 Objective

To compare the clinical efficacy of sustained release vaginal progesterone geland intramuscular progesterone in hormone replacement FET cycles. The primary outcome measure was live birth rate and the secondary outcome measures were clinical pregnancy, miscarriage, ectopic pregnancy and implantation rates.

# 4 Flow chart and visit

## 4.1 Flow chart

Informed consent and screening (menstruation on day 1~3):

- Medical history review
- ultrasound and physical examination

Screening failed

Hormone replacement protocol:

- Estradiol valerate 4 mg/d was administrated on day 3 of menstruation for 10 days;
- 10 days after administration, B-mode ultrasound examination was conducted. If endometrial thickness <7mm, increasing the dose of estradiol valerate to 6-8mg/d;
- If endometrial thickness was ≥7mm, progesterone would be administrated on day 0 to promote endometrium to secretory phase.

Endometrial secretory transformation on day 0

Excluded before random allocation:

Endometrial thickness <7mm

Random allocation

Group A

- Sustained release vaginal progesterone gel 90mg/d
- Dydrogesterone 20mg/d
- Estradiol valerate 4-8mg/d
- Day3 FET

Group B

- Intramuscular progesterone 40mg/d
- Dydrogesterone 20mg/d
- Estradiol valerate 4-8mg/d
- Day3 FET

Determination of pregnancy:

14 days after transfer, blood β-hCG was determined to check whether biochemical pregnancy achieved.

Pregnancy

No pregnancy

- Sustained release vaginal progesterone gel or intramuscular progesterone, dydrogesterone and estradiol valerate were withdrawn.
- Sustained release vaginal progesterone gel or intramuscular progesterone, dydrogesterone and estradiol valerate were administrated continually at the same dose.
- 5 weeks after transfer, vaginal B-mode ultrasound examination was conducted. If gestational sac and fetal heart were observed, it is defined as clinical pregnancy.
- Sustained release vaginal progesterone gel or intramuscular progesterone, dydrogesterone and estradiol valerate were withdrawn.
- 6 weeks after transfer, oral estradiol valerate was withdrawn.
- 8 weeks after transfer, sustained release vaginal progesterone gel or intramuscular progesterone was withdrawn.
- 10 weeks after transfer, dydrogesterone was withdrawn.
- Letter or telephone follow-up was performed to investigate delivery and birth outcomes.

## 4.2 Screening visit

On day 1-3 of menstruation, investigators visited patients with FET in this week, and patients had completed IVF / ICSI cycles. The detailed information had recorded. During the visit, the following items should be completed:

1. Patients signing informed consent form;
2. Reviewing medical history, such as whether there are underlying diseases and other drug use, infertility and gynecological history, and menstrual history;
3. Physical examination, including vital signs, height, weight, gynecological examination (if the examination had carried out within six months, only results were required to record);
4. Examination of the uterus and ovaries using transvaginal ultrasound;
5. Inspecting medical history to confirm whether patients satisfy exclusion criteria, including whether there are d3 frozen embryos, blood routine, hepatic and renal function, blood coagulation series, routine urine, infectious diseases, cervical smears, electrocardiogram and chest radiograph examinations.

## 4.3 Visit on the day of endometrial secretory transformation

During the visit, the following items should be completed:

1. Recording endometrial thickness, days of administration estradiol valerate and completion of the corresponding forms;
2. Excluding patients with endometrial thickness <7mm;
3. Asking patients whether any adverse events had occurred, if any, adverse event reporting form should be completed;
4. Patients randomized allocation.

## 4.4 Visit on the day of embryo transfer

The information of embryos transfer was recorded and the corresponding form was completed. Patients were asked whether any adverse events had occurred, if any, adverse event reporting form should be completed.

## 4.5 Visit on the day of determination of biochemical pregnancy

14 days after embryos transfer, blood β-hCG was determined to inspect whether biochemical pregnancy achieved and the corresponding form was completed. Patients were asked whether any adverse events had occurred, if any, adverse event reporting form should be completed.

## 4.6 Visit on the day of evaluation of clinical pregnancy

5 weeks after transfer, vaginal B-mode ultrasound examination was conducted in blood β-hCG positive patients. If gestational sac and fetal heart were observed, it is defined as clinical pregnancy. Patients were asked whether any adverse events had occurred, if any, adverse event reporting form should be completed.

## 4.7 Visit of delivery outcomes

2 weeks after expected date of confinement, telephone follow-up was performed to ask gestational age, mode of delivery, neonatal quantity, weight, with or without birth defects, and the corresponding form was completed. Patients were asked whether any adverse events had occurred, if any, adverse event reporting form should be completed.

# 5 Design

## 5.1 Overall design

This study was a single-center, prospective, randomized (1: 1) clinical trial. The comparison of live birth rates between sustained release vaginal progesterone gel and intramuscular progesterone in hormone replacement FET cycles was carried out. 1500 patients were recruited and randomly divided into two groups. Patients in group A were received sustained release vaginal progesterone gel and in group B received intramuscular progesterone.

## 5.2 Randomization

1. Personnel who are not involved in this study operated computer to generate random allocation table and 1500 random numbers were randomly divided into two groups in the proportion of 1:1 (for example: random number 1, group A, sustained release vaginal progesterone gel group).

2. After the confirmation of patients involved in the study, investigator operated computer to generate random number on the day of endometrial secretory transformation in hormone replacement FET cycles and the group that patients belonging to was found in the random allocation table. Patients in group A were received sustained release vaginal progesterone gel and in group B received intramuscular progesterone.

3. Both investigator and patients knew the grouping situation on the day of endometrial secretory transformation. For each visit, investigator will inform the patients how to administrate medicine and next visit date.

## 5.3 Protocol of frozen embryo hormone replacement

Estradiol valerate (Progynova, Germany Bayer) 4mg/d, orally, was administrated on day 3 of menstruation for 10 days. 10 days after administration, B-mode ultrasound examination was conducted. If endometrial thickness <7 mm, increasing the dose of estradiol valerate to 6-8 mg/d. If endometrial thickness was ≥ 7 mm, progesterone would be administrated to promote endometrium to secretory phase.

## 5.4 Endometrial secretory transformation and luteal phase support

Group A: On the day of endometrial secretory transformation (D0), patients were administrated sustained release vaginal progesterone gel 90mg/d, dydrogesterone 20mg/d orally and estradiol valerate 4-8 mg/d. Embryo with top score on D3 FET was administrated at the same dose continually to the 14 days after transfer. The number of transferred embryos was defined in accordance with standards of Ministry of Health. For subjects <35 years old with first embryo transfer, 2 embryos were allowed to transfer at most; for subjects ≥ 35 years old or with re-transfer, 2-3 embryos were allowed.

Group B: On the day of endometrial secretory transformation (D0), patients were administrated intramuscular progesterone 40 mg/d, dydrogesterone 20mg/d orally and estradiol valerate 4-8 mg/d. Embryo with top score on D3 FET was administrated at the same dose continually to the 14 days after transfer. The number of transferred embryos was defined in accordance with standards of Ministry of Health. For subjects <35 years old with first embryo transfer, 2 embryos were allowed to transfer at most; for subjects ≥ 35 years old or with re-transfer, 2-3 embryos were allowed.

## 5.5 Pregnancy outcome follow-up

14 days after transfer, blood β-hCG was determined to check whether biochemical pregnancy achieved. For patients with positive hCG, sustained release vaginal progesterone gel or intramuscular progesterone, dydrogesterone and estradiol valerate were administrated continually at the same dose. 5 weeks after transfer, vaginal B-mode ultrasound examination was conducted. If gestational sac was observed, it is defined as clinical pregnancy. 6 weeks after transfer, estradiol valerate was withdrawn. 8 weeks after transfer, sustained release vaginal progesterone gel or intramuscular progesterone was withdrawn. 10 weeks after transfer, dydrogesterone was withdrawn. Letter or telephone follow-up was performed to investigate delivery and birth outcomes. For patients with negative hCG, progesterone, dydrogesterone and estradiol valerate were withdrawn.

## 5.6 Endpoint

The primary outcome measure was live birth rate and the secondary outcome measures were included clinical pregnancy rate, miscarriage rate, implantation rate and so on.

# 6 Subjects selection

Inclusion criteria:

1. Date from September 1^st^, 2010 to January 31^st^, 2013
2. Hormone replacement FET cycles;
3. Subjects with age ≥20 and ≤40 years old;
4. With d3 frozen embryos;
5. Endometrial thickness ≥7mm on the day of secretory transformation;
6. Agreed to sign informed consent form。

Exclusion criteria were in hormone replacement FET cycles

1. Patients with uterine disorders, such as uterine malformations (unicornuate uterus, bicornuate uterus, mediastinal uterus, double uterus), adenomyosis, submucosal fibroids and intrauterine adhesion;
2. Suffered 3 or more spontaneous abortions (including biochemical pregnancy);
3. Embryo transfer in patients failed for 3 or more times (including biochemical pregnancy);
4. Patients not suitable for assisted reproductive technology;
5. Patients that were taking medication or treatment having influence on reproductive and metabolic function, including diabetes drugs, anti-hypertensive drugs (diazoxide, ACEI inhibitors, calcium channel blockers), Chinese herbal medicine and acupuncture;
6. Endometrial thickness <7mm on the day of secretory transformation;
7. Patients who can’t comply with the study protocol.

# 7 Physical examination

Physical examination was conducted and patient's vital signs (temperature, heart rate, respiration and blood pressure), height, weight, and gynecological examination were recorded. Patients with elevated blood pressure (≥160/100) were measured again 15 minutes after the sit-in.

# 8 Transvaginal ultrasound examination

Uterine size, whether there is fibroid and its size, uterine abnormalities (uterine malformations, adenomyosis, endometrial echogenic mass), endometrial thickness, size and shape of the ovaries were inspected. Ovary size was determined by measuring the maximum plane in two dimensions.

# 9 Outcome measures

The primary outcome measure was live birth rate and the secondary outcome measures were included clinical pregnancy, miscarriage, ectopic pregnancy and implantation rates.

# 10 Duration

From September 1^st^, 2010 to January 31^st^, 2013, patients who received hormone replacement FET cycles at Reproductive Medicine Center of Renji Hospital and satisfied inclusion criteria were enrolled in the study. 10 months after transfer, patients were followed-up to investigate birth outcomes. From enrollment to follow-up completed, it would take 28 months (from September 1^st^, 2010 to December 31^st^, 2013).

# 11Data statistics

## 11.1 Estimation of sample size

Yanushpolsky, et al reported in a RCT study that ongoing pregnancy or live birth rates in sustained release vaginal progesterone gel and intramuscular progesterone groups were 45.2% and 42.2%, respectively, in FET cycles ^[3]^. Two domestic studies were reported respectively that clinical pregnancy rate in 155 patients who received intramuscular progesterone in hormone replacement FET cycles to promote endometrium to secretory phase was 34.8% and in 315 patients was 23.5% ^[8-9]^. Assuming that live birth rate in sustained release vaginal progesterone gel group in hormone replacement FET cycles was 10% higher than that in intramuscular progesterone group (40% in sustained release vaginal progesterone gel group and 30% in intramuscular progesterone group), 675 cycles were required to reach 90% statistical power for each group. Considering about 10% of dropout rate, 750 cycles were required for each group and a total of 1500 cycles for 2 groups.

Calculation of sample size

Significance level α: 0.01

1-β: 0.90

Live birth rate in group A: 0.40

Live birth rates in group B: 0.30

Ratio: 1:1

The minimum sample size: 675 cycles for each groups and a total of 1350 cycles for 2 groups.

Considering about 10% of dropout rate, 750 cycles were required for each group and a total of 1500 cycles for 2 groups.

## 11.2 Statistical methods

Differences in live birth rates between the two groups were discussed using intentional analysis. Data were compared using statistical software SPSS 19.0. Results were expressed as mean ± standard deviation (X ± SD) or percentage (%).Count data was analyzed using chi-square test and measurement data using T-test.

# 12 Adverse event reports

## 12.1 Risks and discomfort

This study doesn’t have any additional risks and discomfort. The following table lists the possible risks and discomfort during the study.

| **Events** | **Risks and discomfort** |
| --- | --- |
| Thawing embryo | Embryo recovery failed and 95% of recovery success rate |
| Embryo transfer | Infection |
| Transvaginal ultrasound | Discomfort in abdominal or pelvis |
| Administration of sustained release vaginal progesterone gel | Vaginal itching and other symptoms |
| Intramuscular progesterone | Injection pain and injection site may occur abscess and panniculitis and allergic reactions |
| Ectopic pregnancy | Ectopic pregnancy may be required. If the treatment is delayed, parts of pregnancy may rupture leading to intraperitoneal blooding and even shock or death in severe conditions. |
| Multiple pregnancies | Fetal reduction may be performed, increasing the risks of pregnancy complications, fetal malformations and preterm birth. |
| Infertility treatment | Anxiety and bad mood at varying degrees |

Patients will be randomly assigned to a group and the curative effect in this group may be worse than in the other group. But, investigator will try to avoid the occurrence of such risks and discomfort. If that happens, patients will be treated promptly and charged routinely. This study does not provide any free treatment and financial compensation.

## 12.2 Definition of adverse event

Adverse event refers to the subjects participating in the study suffered any unexpected or adverse medical events, whether or not related to the study intervention. Adverse events may be any of the following:

- Signs or symptoms, including side effects of drugs;
- Abnormal laboratory parameters;
- Changes in vital signs, physical examination or test results;
- Frequency or extent (deterioration) of previous symptoms or diseases before recruitment increasing during the study.

Adverse events in this study do not include:

- Previous symptoms or diseases before recruitment without worsen during the study;
- Normal reactions related to pregnancy

Serious adverse events include any of the following occurred during the study:

- Death
- Life-threatening (there is a risk of immediate death)
- Severe or permanent disability
- Hospitalization required or prolonged
- Neonatal death at 6 weeks postpartum
- Newborns with severe congenital anomaly or birth defect
- Investigator regarding the events as serious events

## 12.3 Adverse event log

Adverse events caused by intervention, observations suspected to have a causal relationship with intervention, adverse events reported by subjects (whether or not serious) and abnormal detection parameters should be recorded in the case report form. A diagnostic rather than a symptom should be recorded. All adverse events will be recorded in detail with the purpose of 1) providing sufficient information to judge adverse events (such as whether the event should be classified as serious adverse events); 2) assessing the causality of adverse events between the intervention and trial. Each adverse event (or its sequelae) or abnormal detection parameters will always be followed up to its cure, alleviate or stabilize at an investigator acceptable level.

## 12.4 Causality and severity assessment

Principal investigator will promptly review records of adverse events and abnormal detection parameters to determine 1) whether the abnormal detection parameters should be classified as an adverse event; 2) whether there are evidences to prove adverse events associated with the study intervention; 3) whether it is serious adverse event. Each adverse event is logged as "probably relevant" or "may not be relevant." If the causality is ambiguous, it is considered as "probably relevant".

The degree of each adverse event should be evaluated and recorded as one of the following:

- Mild: Subjects with symptoms or signs may inform investigator. Subjects had symptoms or signs but easily tolerated.
- Moderate: Symptoms affect the normal activities and treatment may be required.
- Severe: Patients always feel these symptoms influencing their daily lives and medical interventions are needed.

## 12.5 Immediate action following a serious adverse events

During the period of informed consent form signed to all subjects completing the trial (the end of last follow-up), if any serious adverse events occurred, investigators should fill "serious adverse event reporting form" immediately, call and send fax or e-mail to relevant safety monitor person within 24 hours. Contacts as follows:

Tel: 021-68383364

Fax: 021-68383364

E-mail: rjluqi@hotmail.com

Unless the subjects were lost to follow up, investigators should monitor and follow up all serious adverse events found in the study to symptoms stabilize or final results of adverse events clarity. Meanwhile, investigator should provide the necessary treatment and follow-up. Any new events (or follow-up information) occurred in the previous reported serious adverse events, its reporting methods and deadlines are the same as initial report.

Investigators should report adverse reactions to the Shanghai Adverse Drug Reaction Monitoring Center according to the requirements of *Management measures on adverse drug reaction reporting and monitoring*.

# 13 Ethics

The study has been approved by Ethics Committee of Renji Hospital Shanghai Jiaotong University School of Medicine (Reviewed by Ethics Committee of Renji, No.[2010] 067).

# 14 References

[1]Tavaniotou A, Smitz J, Bourgain C,et al. Comparison between different routes of progesterone administration as luteal phase support in infertility treatments. Hum Reprod Update, 2000,6(2):139–148.

[2] Cicinelli E, de Ziegler D, Bulletti C, et al. Direct transport of progesterone from vagina to uterus. Obstetrics & Gynecology, 2000, 95(3): 403-406.

[3] YanushpolskyE, Hurwitz S, Greenberg L, et al. Crinone vaginal gel is equally effective and better toleratedthan intramuscular progesterone for luteal phase support in in vitrofertilization-embryo transfer cycles: a prospective randomized study. Fertility and Sterility, published online March 29, 2010

[4] YanushpolskyE, Hurwitz S, Greenberg L, et al. Patterns of luteal phase bleeding in in vitrofertilization cycles supplemented with Crinone vaginalgel and with intramuscular progesterone—impact ofluteal estrogen: prospective, randomized study andpost hoc analysis . Fertility and Sterility, published online May 26, 2010

[5]Dal Prato L, Bianchi L, Cattoli M, et al. Vaginal gelversus intramuscular progesterone for luteal phase supplementation: a prospectiverandomized trial. Repro Biomed Online 2008, 16(3):361–367.

[6]Kahraman S,Karagozoglu SH,Karlıkaya G, et al.The efficiency of progesterone vaginal gel versusintramuscular progesterone for luteal phasesupplementation in gonadotropin-releasing hormoneantagonist cycles: a prospective clinical trial. Fertility and Sterility, 2010, 94(2) : 761－620.

[7]Jobanputra K, Toner JP,Denoncourt R, et al.Crinone 8% (90 mg)given once daily for

progesterone replacement therapy in donoregg cycles.Fertility and Sterility, 1999, 72(6) : 980－984.

[8] Li Yufeng, Gui Jin, Zhang Hanwang, et al. Comparison of 3 endometiral preparation for frozen-thawed embryo transfer. Journal of Reproduction and Contraception, 2009;29(2):113-116.

[9] He Ling, Liu Fenghua, Long Xiaolin, et al. Compare the outcome of frozen-thawed embryo transfer between in natural cycles and artificial cycles. Chinese Journal of Practical Gynecology and Obstetrics, 2009;25(9):694-696.

# Annex 1: Informed consent form

# Annex 2: Case report form (CRF)

**Appendix 1: Informed Consent Form**

**Informed Consent Form · Informed Consent Page**

Dear Patients:

Doctors have confirmed diagnosis that you need to realize pregnancy by FET (Frozen-thawed Embryo Transfer). We will invite you to participate in a study integrated in the STCSM Natural Science Foundation Program (Project number: 08zr1413400). The research program was verified by the Ethics Committee, Renji Hospital Shanghai Jiaotong University School of Medicine, and the clinical study was approved.

Before you decide whether to participate in the study, please read the following as carefully as possible. It can help you understand the reasons for the conduct of this study, the study procedures and duration, the benefits, risks and discomforts it may bring to you after participating in this study. If you prefer, you may also discuss with your relatives and friends, or ask the doctor to give an explanation to help you make a decision.

**I. Background and Objective**

**1.1 Disease burden and treatment status**

With the development of the society, infertility has become the third largest disease that affects humankind, following tumors and cardiovascular diseases. In China, about 10%-15% of child-bearing couples have fertility problems, and there is a trend of increase year by year. The usual "Test tube baby" technology is to conduct fresh embryo transfer after ovarian stimulation and oocyte retrieval. However, the high levels of estrogen generated in ovarian stimulation process may affect the embryo implantation process, and there is also risk of OHSS (ovarian hyperstimulation syndrome), usually manifested as abdominal distension, ascites, ovarian increase, etc. Therefore, it is required to keep all embryos frozen after oocyte retrieval, and re-conduct FET after the ovarian size and estrogen level have returned to normal. Fresh cycle endometrium is not suitable for transplantation, or there is also need for those patients who have frozen embryos and remain unpregnant after transfer to receive FET.

The hormone replacement frozen-thawed embryo transfer cycles is widely used, due to its simple monitoring, flexible control, low cycle cancellation rate and other advantages. However, as there are no follicular development and ovulation in hormone replacement cycles, it is required to artificially use the progestational hormone to conduct the secretory phase transformation of endometrium to accept the embryo implantation and continued development. Natural progesterone is the most commonly-used corpus luteum support drug, primarily intramuscular injection in the past in China. As a kind of micronized natural progesterone vaginal preparations, crinone utilizes the targeted uterine first pass effect, and enables higher uterine local concentration, lower proportion of absorbed into the bloodstream, and effectively-reduced risk of systemic side effects. At present, there is still no final conclusion as to which medication has better effects.

**1.2 The purpose of this study**

It is planned to compare the clinical outcomes between the application of sustained release vaginal progesterone gel and the application of intramuscular progesterone during the hormone replacement frozen-thawed embryo transfer cycles, and subsequently decide which medication can achieve higher clinical pregnancy and live birth rate.

**1.3 Study participating units and the number of participating patients**

This study will be conducted at Renji Hospital Shanghai Jiaotong University School of Medicine, and approximately 1500 women will be included in the study.

**II. Who should not participate in the study?**

Three or more spontaneous abortions (including biochemical pregnancy abortion);

Embryo transplant failure for more than three times (including biochemical pregnancy)

The patients suffer from abnormal uterine diseases, including uterine malformations (unicornuate uterus, mediastinal uterus, double uterus, bicornuate uterus), adenomyosis, submucous myoma, intrauterine adhesion and so on.

The patients suffer from the diseases not suitable for the application of ART (assisted reproductive techniques)

**III. What required to be done if you have participated in the study?**

1． Before you are chosen to participate in the study, the doctor will ask, record your medical history, and conduct pre-pregnancy checks associated with the test-tube baby operation.

As a qualified participant, you can be volunteered for the study, and sign the informed consent form.

If you do not want to participate in the study, we will undertake the treatment according to your wishes.

2. If you voluntarily participate in the study, the treatment will be carried out according to the following steps:

All treatments you will receive belong to the routinely-performed clinical measures, and all trial subjects will be randomly divided into two groups at the endometrial conversion date of frozen-thawed embryo transfer cycles. For the group A, the patients will be treated with the sustained release vaginal progesterone gel (Crinone, Switzerland Merck-Serono) 90mg/d. Meanwhile, the patients will be instructed to orally take dydrogesterone (Duff pass, USA Abbot) 10mg, bid, estradiol valerate (progynova, Germany Bayer) 4-8mg/d. If the patients become pregnant, it is required to keep the sustained medication with dosage remaining unchanged, until the cessation of orally-administrated estradiol valerate 6 weeks after transplantation, cessation of sustained release vaginal progesterone gel 8 weeks after transplantation, and cessation of orally-administrated dydrogestrone 10 weeks after transplantation. For the group A, the patients will be treated with intramuscular progesterone 40mg/d. Meanwhile, the patients will be instructed to orally take dydrogesterone 10mg, bid, estradiol valerate 4-8mg /d. If the patients become pregnant, it is required to keep the sustained medication with dosage remaining unchanged, until the cessation of orally-administrated estradiol valerate 6 weeks after transplantation, cessation of intramuscular progesterone 8 weeks after transplantation, and cessation of orally-administrated dydrogestrone 10 weeks after transplantation.

14 days after transplantation, it is arranged to check the bllodβ-hCG and determine whether the biochemical pregnancy exists or not. The patients with hCG positive are required to go to hospital 5 weeks after the transplantation, and clinical pregnancy can be ascertained if gestational sac and fetal heart are detected by B-scan. Letters or telephone calls are followed by childbirth and new-born baby conditions.

3. Other Matters requiring your cooperation

You must come to see the doctor as per the follow-up survey time arranged between you and the doctor (during the follow-up phase, the doctor may understand your situation by telephone calls or letters). Your follow-up survey is very important, because the doctor will determine whether your treatment really works, and provide timely guidance for you.

You must take medicine by following the doctor’s instruction, and you are also required to promptly and objectively fill in your medication records. At each follow-up survey, you must return the unused drugs and their packages, and bring about other drugs, including those must-be-taken ones for treatment of your other concomitant diseases.

During the study period, you are not permitted to take other progesterone drugs.

If you need other treatments, please contact in advance with your doctor.

**IV. Possible benefits from the participation of this study**

You will be randomly assigned to either a treatment group, and the final results may indicate that your treatment is more effective than the other one group and other treatments, or the incidence rate of adverse reactions may be even lower. The results of this study will be of guideline value to the treatments of those patients who have the same situation with your in the future, and will also help promote the understanding of medical field on the corpus luteum support in hormone replacement frozen-thawed embryo transfer cycles.

**V. Possible adverse effects, risks & discomforts, inconveniences caused by the participation of study**

Compared with those patients usually accept frozen-thawed embryo transfers, the participation of this study may not increase any additional risks. If you experience any discomforts during the study, new changes in the state of illness, or any other unforeseen circumstances, whether or not related to the study, you should promptly notify your doctor, and he/she will make corresponding judgments and provide proper medical treatments.

During the study period, your are required to go to hospital to make follow-up survey and receive some checks, which may occupy some of your time, or bring you some troubles or inconveniences.

**VI. Related costs**

All the costs incurred from this study process shall be borne by the patients. Doctors will make every effort to prevent and treat those damages possibly caused by this study. Once adverse events or injuries have occurred, this study will provide appropriate treatment measures, but you need to pay for these treatments, and the costs will be charged according to the conventional charging standards. Therefore, this study shall not provide financial compensation or free medical treatment.

**VII. Will the personal information kept confidential?**

Your medical records (medical records of this study/CRF, laboratory reports, etc.) will be kept intact at the hospital where you received treatment. The doctor will record the laboratory test results in your medical records. Researchers, ethics committee and the pharmaceutical supervisory & administrative departments shall be allowed to refer to your medical records. Your personal identity will not be disclosed in any public reports related to the results of this study. We will make every effort within the scope permitted by law to protect the privacy of your personal medical information.

**VIII. How to get more information?**

You can ask any questions about this study at any time and get corresponding answers. If there are any important new information during the study process that may affect your willingness to continuously participate into this study, your doctor will keep you informed.

**IX. You are permitted to voluntarily choose to participate in the study and withdraw from the study**

It is completely up to you whether or not to participate in the study. You may refuse to participate in the study, or withdraw from this study at any time, which will not affect the relationship between you and the doctor, as well as your treatment or interest losses in other aspects.

In consideration of your best interests, doctors and researchers may terminate, at any time during the study process, your continued participation in this study.

If you withdraw from the study for any reason, you may be asked about the situation regarding your use of the study drug. You may also be asked to carry out laboratory tests and physical examinations, if the doctor considers it necessary.

**X. What should I do right now?**

Whether or not to participate in this study shall be determined by you (and your family).

Before you make a decision to participate in the study, please ask your doctor about relevant questions to the maximum extent possible.

Thank you for reading the above materials. If you decide to participate in this study,

Please tell your doctor, and he/she will arrange all matters relating to study.

Please properly keep this material.

**Informed Consent Form/Consent Signature Page**

**Name of Clinical Research Program:** A comparison of live birth rates between sustained release vaginal progesterone gel and intramuscular injection of progesterone in hormone replacement frozen-thawed embryo transfer cycles: a randomized prospective study in Chinese population

**Project Undertaking Unit**: Renji Hospital Shanghai Jiaotong University School of Medicine

**Project Specification No.** 08zr1413400

**Declaration of Consent**

I have read the above introduction about this study, got the opportunity to discuss with the doctor about this study and ask some questions. All my questions have been answered in a satisfactory manner.

I know about the risk and benefits possibly generated from the participation of this study. I know that my participation in this study is on voluntary basis, and I confirm that I have a plenty of time to consider this issue, and realize that:

- I can consult the doctor at any time for more information.
- I can withdraw from this study at any time, without the risk of discrimination or retaliation, and my medical treatment and interests will not be affected.

I also know, if I drop out this research, especially when I withdraw from the study for the reason of drugs, I should tell my changes in condition to the doctor, complete the appropriate physical examination and physical & chemical checks, which will be very favorable to the entire study.

If I need to take any other medication due to the changes in condition, I will seek the medical advice in advance or truthfully tell the doctor after the event.

I agree that the representative from the drug regulatory authority or the sponsor may refer to my study records.

I will receive a copy of informed consent form signed and marked with the date.

Finally, I have decided to agree to participate in this study, and try my best to follow the doctor's advice.

Patient's Signature: M/D/Y

Contact Phone:

I confirm that I have explained the details of this test to the patient, including her rights, possible benefits and risks, and I have also given her a signed copy of the informed consent form.

Patient's Signature: M/D/Y

Doctor’s work phone:：

#

**Appendix 2:** **Case Report Form (CRF)**

A comparison of live birth rates between sustained release vaginal progesterone gel

and intramuscular injection of progesterone in hormone replacement frozen-thawed

embryo transfer cycles: a randomized prospective study in Chinese population

**Case Report Form (CRF)**

**August 15, 2010**

**Research Program：** A comparison of live birth rates between sustained release vaginal progesterone gel and intramuscular injection of progesterone in hormone replacement frozen-thawed embryo transfer cycles:

**Primary Screening Test Form (Page 2 to 5)**

| **Patient Name：** | **Medical record number #：** |
| --- | --- |
| **Study No.：** | **Time of Signing the Letter of Consent：YTD** |
| **Date of Birth：YTD** | **Age：Year** |
| **Tel 1：**  **Level of Education：** | **Tel 2：** |

**Medical History**

1. Do you have a previous history of any one of the following diseases or discomforts?

1a. any one of the following diseases or conditions □ No

□ Yes

Whether or not take medicines for current treatment？□Yes □No

(Please record your therapeutic medication: ____________________)

Is the disease control satisfactory？□Yes □No

1b. Thyroid disease□ No

□ Yes

Whether or not take medicines for current treatment？□Yes □No

(Please record your therapeutic medication: ____________________)

Is the disease control satisfactory？□Yes □No

1c. Diabetes (unrelated to pregnancy) □No

□Yes

Whether or not take medicines for current treatment？□Yes □No

(Please record your therapeutic medication: ____________________)

Is the disease control satisfactory？□Yes □No

1d. Diagnosed with symptoms of heart disease □No

□Yes

After medical consultation , is your heart function able to withstand pregnancy？□Yes □No

1e. History of deep vein thrombosis □No □Yes

1f. Confirmed / suspected malignancy at cervical, breast or endometrial and other parts？□No □Yes

1g. Have you suffered from tuberculosis over the past six months？□No □Yes

2. Are you taking the following medications or receiving treatments as follows?

□Chinese medicinal herb / Chinese patent drug

□Multi-vitamin/folic acid

□Acupuncture and moxibustion

□None of the above

**Menstrual history**

1. Your age of menarche？ __ __year old

2. How many days between two menstruation start dates？The longest period： __days；The shortest period：__days

3. Have you used gynecological drugs to regulate the menstrual cycle?

□Yes □No

What kind of gynecological drugs have you ever used to regulate the menstrual cycle?

□Oral contraceptive pill

□Progesterone

□Traditional Chinese medicine

□Artificial cycle

4. Date of last menstrual period：//（Year / Month / Day）

**History of infertility**

1. How long is your pregnancy test？__ years
2. What is the cause of infertility diagnosed by your attending physician?

2a. Your diagnosis？（Please check all that apply）

□Ovulation failure

□Tubal factor

□Male factor

□Other factors or unknown causes

3. Have you received progesterone treatment？□No（if no, jump to the gynecological history）

□Yes

□Insulin Sensitizer（Metformin, rosiglitazone, or others）

□Induced ovulation treatment

□Clomiphene citrate ( France to Portland , snow Lanfen )

□Letrozole

□Gonadotropin ( including rFSH，HMG）

□Others

□Intrauterine insemination（IUI）

□Traditional Chinese medical science

□Others（please specify）：________________

**Gynecological history**

1. Have you ever received gynecologic operation in the past？□No □Yes

- Ovarian Cyst excision
- Unilateral oophorectomy
- Ovarian drilling technique under laparoscopy（LOD）
- Hysteromyomectomy
- Endometriosis -related surgery
- Infertility-targeted hysteroscopy laparoscopy combined exploratory operation
- Operation for treatment of hydrosalpinx
- Ovarian tumor surgery
- Others

1. GPAL
2. Is your medical history of recurrent spontaneous abortion (Including biochemical pregnancy miscarriage)≥3 times in the past? □Yes □No

**Physical examination & measurement**

Date of examination：//（Y/M/D日）

1. Height cm

2. Weight kg

3. Blood Pressure：/mmHg （Systolic/diastolic）

4. Gynecological examination：□Normal □Abnormal（Please specify：______________________）

**Transvaginal ultrasound B results**

1. Date of examination：//（Y/M/D日）

Menstrual Cycle：Which day

1. Size of the uterus：cm ×cm，

Hsteromyoma：□ No □Yes

□Intramural

□Submucosal

□Subserosal

The number of fibroids：□Single □Multiple

Average diameter of the largest myoma： cm

For other uterine abnormalities, please describe：______________________

Endometrial thickness：cm

Size of right ovary：cm ×cm，oophoritic cyst：

Size of left ovary：cm ×cm，oophoritic cyst：

**Security primary screening test results (valid within the past year):**

| **Test Items** | **Date of Examination** | **Results（Normal√；abnormal × add the details）** |
| --- | --- | --- |
| Blood routine examination |  |  |
| Liver function |  |  |
| Renal function |  |  |
| Coagulation function |  |  |
| Infectious disease screening（HBV, HCV, HIV, syphilis） |  |  |
| Routine urine test |  |  |
| TCT/LCT |  |  |
| Electrocardiogram ECG |  |  |
| DR or |  |  |

**Into-group conditions（****Answer requiring to be "YES"）**

1. The patient's age at the time of informed consent≥20y and ≤40y？□Yes □ No
2. Is the patient have d3 frozen embryos？□Yes □ No

**Exclusion criteria（Answer requiring to be "No"）**

1. Whether or not the patient is diagnosed with uterine abnormalities（Including uterine malformations, adenomyosis, submucous myoma , uterine adhesions）？□No □Yes
2. Whether or not the patient has the medical history of three or more than three times of recurrent spontaneous abortions (Including biochemical pregnancy miscarriage)? □No □Yes
3. Whether or not the patient has the medical history of three or more than three times of embryo transfer failures (Including biochemical pregnancy miscarriage)? □No □Yes
4. Whether or not the patient is taking or receiving drugs or treatments that may affect the reproductive and metabolic function？□No □Yes
5. Based on the above history review and preoperative examination, Whether or not the assisted reproductive technology and pregnancy contraindications exist? □No □Yes

Research Program：A comparison of live birth rates between sustained release vaginal progesterone gel and intramuscular injection of progesterone in hormone replacement frozen-thawed embryo transfer cycles:

**Inspection Visit Form for** **Endometrium Secretory Phase Transformation Date (page 6)**

**Menstrual type before the preparation of hormone replacement endometrial cycles:**

□Spontaneous menstruation □Progesterone withdrawal □ OCP withdrawal

**Estradiol valerate treatment**

Number of days of estrogenic hormone therapy：__ __days

**Endometrial transformation date B-scan results：**

The total number of follicles：14 - 18 mm ( >=14mm且<18mm) piece; >=18 mm piece

Endometrial thickness：mm，typing _____.

**Hormone level of endometrial transformation date：**

E2: ______pg/ml ; LH: ______IU/L; P ______ng/dl

**Endometrial** **transformation**

Date of transformation：__ ____ __/__ __/__ __ （Y/M/D）

**Random front** **scaling conditions（If any one answer is "yes "，the patient should withdrew from the study）**

Endometrial thickness＜7mm？□Yes □ No

**Randomized grouping**

1. Whether or not the patient complies with the random grouping conditions？□ Yes □ No

2. Whether or not the patient agrees to be randomly grouped？□ Yes □ No

3.Date of Randomization：__ ____ __/__ __/__ __（Y/M/D）

4. The patient will be randomly assigned to：

□Group A（sustained release vaginal progesterone gel group）□Group B（Intramuscular progesterone group）

Research Program：A comparison of live birth rates between sustained release vaginal progesterone gel and intramuscular injection of progesterone in hormone replacement frozen-thawed embryo transfer cycles:

**Inspection Visit Form for Embryo Transfer Date** **(page 7)**

**Frozen-thawed** **embryo transfer**

1. Endometrial thickness before transfer：mm；

2. The number of embryos transfer：______

3. The number of remaining frozen-thawed embryos：______

4. Day-old of remaining frozen-thawed embryos：

□D3 □D5

5. Reasons for not-performed embryo transfer：

□Thaw failure of embryos，no available embryo transfer

□Uterine factor

□Others

Research Program：A comparison of live birth rates between sustained release vaginal progesterone gel and intramuscular injection of progesterone in hormone replacement frozen-thawed embryo transfer cycles:

**Inspection Visit Form for Determination of Biochemical Pregnancy (page 8)**

1. Pregnancy test__ days after embryo transfer
2. Blood HCG test value：______IU/L；
3. Whether or not the patient has obtained biochemical pregnancy？□ Yes □ No

Research Program：A comparison of live birth rates between sustained release vaginal progesterone gel and intramuscular injection of progesterone in hormone replacement frozen-thawed embryo transfer cycles:

**Inspection Visit Form for the Date of Clinical Pregnancy Assessment**

**(Page 9)**

1. This B –scan shall be conducted within ___days after the embryo transfer.
2. Whether or not the patient has obtained the clinical pregnancy?

□Yes

The number of intrauterine gestational sac：

The number of embryos with fetal heart beat：

□If no，the results shall be：

□Biochemical pregnancy miscarriage

□Right fallopian tube pregnancy

□Left fallopian tube pregnancy

□CHM

□PHM

□Heterotopic pregnancy

3. EDC of the patient：__ ____ __/__ __/__ __ （Y/M/D）

Research Program：A comparison of live birth rates between sustained release vaginal progesterone gel and intramuscular injection of progesterone in hormone replacement frozen-thawed embryo transfer cycles:

**Inspection Visit Form for Condition of Delivery** **(page 10)**

1. Date of Delivery：__ ____ __/__ __/__ __

2. Gestational age at the childbirth：Pregnant for __ __ Week

3. Vaginal delivery？□Yes □ No

4. Cesarean section？□Yes □ No

5. Number of live births：

6. Stillbirth？ □Yes □ No

Number of stillbirth：

Reasons for stillbirth？（Tick ​​all that apply）□Unspecified

□Birth Defects

□Placental abnormality

□Fetal Growth Retardation

□Infection

□Mother’s disease

□Umbilical cord accident

□Others（Please specify）：_________________

7. Newborn 1 Information

1）. Neonatal sex：□Male □Female

2）. Neonatal birth weight： __ __ __ __ g；

3）. Existence of birth defects：□Yes □ No

8. Newborn 2 Information

1）. Neonatal sex：□Male □Female

2）. Neonatal birth weight： __ __ __ __ g；

3）. Existence of birth defects：□Yes □ No
